# Multi-Omics Analysis Reveals 1-Propanol-Induced Pentadecanoic Acid Biosynthesis in *Yarrowia lipolytica*

**DOI:** 10.3390/biom15111618

**Published:** 2025-11-18

**Authors:** Jiahe Cong, Xin Hu, Dongsheng Lu, Sam C. Kollie, Ahmed A. Elolimy, Juan J. Loor, Zhendong Yang, Mingxun Li, Yongjiang Mao, Zhangping Yang, Huimin Zhang

**Affiliations:** 1 Key Laboratory for Animal Genetics, Breeding, Reproduction and Molecular Design of Jiangsu Province, College of Animal Science and Technology, Yangzhou University, Yangzhou 225009, China; mz120231607@stu.yzu.edu.cn (J.C.); 231902408@stu.yzu.edu.cn (X.H.); mx120240901@stu.yzu.edu.cn (D.L.); mh23096@stu.yzu.edu.cn (S.C.K.); limingxun@yzu.edu.cn (M.L.); cattle@yzu.edu.cn (Y.M.); yzp@yzu.edu.cn (Z.Y.); 2 Department of Integrative Agriculture, College of Agriculture and Veterinary Medicine, United Arab Emirates University, Al Ain 15551, United Arab Emirates; elolimy@uaeu.ac.ae; 3 Department of Animal Sciences & Division of Nutritional Sciences, University of Illinois, Urbana, IL 61801, USA; jloor@illinois.edu; 4 Technology Innovation Center of Meat and Meat Products, State Administration for Market Regulation, Jinan 250101, China; spgc@shandong.cn

**Keywords:** pentadecanic acid, *Yarrowia lipolytica*, 1-propanol, transcriptomics, metabolomics

## Abstract

Pentadecanoic acid (C15:0) is an odd-chain fatty acid (OCFA) with significant health benefits, mainly produced by microbial fermentation. To improve C15:0 production, this study compared the effects of different alcohols on C15:0 production in *Yarrowia lipolytica* CICC1778, identified 1-propanol as the most effective precursor, assessed its optimal concentration, and employed transcriptomic and metabolomic analyses to elucidate the regulatory mechanisms. The results showed that supplementation with 0.5% 1-propanol resulted in a total lipid production of 1.54 g/L in *Y. lipolytica* CICC1778, showing no differences compared with the negative control (NC) group, while C15:0 production increased to 76.68 mg/L, representing a 794.7% increase compared with the NC group. Integrated omics analysis showed that propionylcarnitine was positively correlated with *ADH2*, *ADH1*, *ACADSB*, *ALDH6A1*, and *CAT2*; O-methylmalonylcarnitine was positively correlated with *IVD*, *MCCC2*, *ACADSB*, and *ALDH6A1*; and (R)-leucic acid and 2-hydroxy-3-methylbutyric acid were positively correlated with *IVD*, *BAT2*, *MCCC2*, and *ACADSB* and *ALDH6A1* and *BAT2*, respectively. All of these DEGs and DEMs were upregulated in the alcohol-treated (ALC; supplementation with 0.5% 1-propanol) group. Taken together, supplementation with 0.5% 1-propanol was an effective strategy for enhancing C15:0 production in *Y. lipolytica* CICC1778; 1-propanol underwent dehydrogenation-oxidation and promoted branched-chain amino acid degradation to expand the propionyl-CoA pool, thereby facilitating C15:0 synthesis.

## 1. Introduction

Pentadecanoic acid (C15:0), an odd-chain fatty acid (OCFA), contributes to human health by alleviating anemia, regulating blood lipids, reducing fibrosis, and exerting antioxidant effects [1]. In nature, C15:0 is predominantly found in the adipose tissues and milk fat of ruminant animals, accounting for approximately 1% of total fatty acids [2,3,4]. In contrast, microbial fermentation has been recognized as the most efficient approach for producing C15:0 [5,6,7]. In previous studies, supplementation with different precursors led to C15:0 production of more than 3% in *Rhodococcus opacus* and *Yarrowia lipolytica* [7,8]. In microbial systems, C15:0 is synthesized when propionyl-CoA, rather than acetyl-CoA, serves as the primer unit for fatty acid synthase (FAS) [9]. However, most microorganisms naturally produce negligible amounts of C15:0, and strategies to enhance their biosynthesis remain an active area of metabolic engineering research [7].

*Y. lipolytica* is an oleaginous yeast recognized for its high lipid accumulation capacity, broad substrate utilization, and Generally Recognized As Safe (GRAS) status, making it a promising candidate for efficient C15:0 production [10,11]. Previous studies have demonstrated that supplementation with propionate increased C15:0 production in *Y. lipolytica* [7,9]. Among other propionyl-CoA precursors, 1-propanol causes less disturbance to the medium pH than propionate and avoids the undissociated weak-acid toxicity associated with propionate, thereby exerting a weaker inhibitory effect on cell growth [12]. In yeast, 1-propanol readily traverses the cell membrane and can be oxidized by dehydrogenase systems to propionate, which is subsequently activated to propionyl-CoA, facilitating propionyl-CoA supply [13]. Zhang et al. demonstrated that supplementation with 1-propanol in *Rhodococcus opacus* increased C15:0 production while decreasing the production of even-chain fatty acids (ECFAs) by upregulating alcohol dehydrogenase (*ADH*) and aldehyde dehydrogenase (*ALDH*), thereby enhancing propionyl-CoA supply [8]. Therefore, 1-propanol is a promising substrate to enhance C15:0 synthesis in *Y. lipolytica* and merits further investigation.

Transcriptomic analysis provides a comprehensive view of global gene expression changes, while metabolomic analysis captures the downstream alterations in metabolite levels that reflect cellular physiology [14,15]. Integrated transcriptomic and metabolomic analyses can identify regulatory networks and functional pathways that cannot be elucidated by single-omics approaches, thereby providing a more comprehensive understanding of cellular responses [16,17]. Previous studies employing transcriptomic and metabolomic analyses of *Y. lipolytica* have shown that, under environmental perturbations such as changes in carbon and nitrogen availability, the strain underwent coordinated reprogramming of gene expression and metabolite profiles, thereby redirecting carbon flux toward lipid synthesis [18,19]. However, the molecular mechanisms by which 1-propanol influences carbon flux distribution and C15:0 synthesis in *Y. lipolytica* remain poorly understood. Integrated transcriptomic and metabolomic analyses provide a powerful approach to elucidate the regulatory networks and metabolic pathways involved in this process [17]. Here, this study compared the effects of different alcohols as carbon sources on C15:0 production in *Y. lipolytica* CICC1778, identified 1-propanol as the most effective precursor, subsequently optimized its concentration, and employed transcriptomic and metabolomic analyses to uncover the pathways underlying its stimulatory effect on C15:0 synthesis.

## 2. Materials and Methods

### 2.1. Yeast Strain

*Y. lipolytica* CICC1778 used in this study was obtained from the China Center of Industrial Culture Collection (CICC) and preserved at −80 °C in 20% (*v*/*v*) glycerol.

### 2.2. Cultivation Conditions

*Y. lipolytica* CICC1778 was inoculated into 50 mL YPD medium in 250 mL Erlenmeyer flasks at an inoculum size of 0.3% (*v*/*v*), corresponding to an initial OD_600_ of 0.2 in the culture, and cultivated at 25 °C and 160 rpm until the exponential phase to obtain the seed culture. The YPD medium consisted of 20 g/L glucose, 20 g/L peptone, and 10 g/L yeast extract, with a pH of 6.5 ± 0.2 (Hopebiol, Qingdao, China).

For fermentation, the seed culture was inoculated into 200 mL of fermentation medium in 500 mL Erlenmeyer flasks to an initial OD_600_ of 0.2. The fermentation medium for the negative control (NC) group consisted of 15 g/L glucose, 1.0 g/L NH_4_Cl, 2 g/L yeast extract, 2.5 g/L KH_2_PO_4_, 2 g/L Na_2_HPO_4_, 1 g/L MgSO_4_·7H_2_O, 0.02 g/L MnCl_2_·4H_2_O, 0.02 g/L CaCl_2_·2H_2_O, 5 mg/L FeNaEDTA, and 0.5 mg/L Na_2_MnO_4_·2H_2_O. The fermentation medium of the experimental groups was supplemented with other carbon sources based on the NC group: (1) for investigating the characteristics of growth and lipid production of *Y. lipolytica* CICC1778, the glucose was increased to 25 g/L (C/N ≈ 30, which is generally considered optimal for lipid accumulation by *Y. lipolytica* [20,21,22]); (2) for screening different carbon sources, each group was supplemented with 0.5% (*v*/*v*) of propionate, methanol, ethanol, 1-propanol, 2-propanol, 1-butanol, and 1-pentanol; (3) for screening different 1-propanol concentrations, each group was supplemented with 0.25%, 0.5%, 0.75%, 1.0%, 1.5%, 2.0%, and 2.5% (*v*/*v*) 1-propanol. All medium components were from Macklin (Shanghai, China).

### 2.3. Determination of Biomass

The optical density (OD) of cultures was measured at 600 nm using a SPARK microplate reader (Tecan, Zurich, Switzerland), with sterile medium as the blank control. If the measured OD_600_ exceeded 0.8, the sample was diluted between 0.2 and 0.8. Dry cell weight (DCW) was determined as described by Park et al. [7]. Briefly, 20 mL of culture was transferred into pre-dried, constant-weight centrifuge tubes and centrifuged at 9000× *g* for 10 min to remove the medium components. The DCW was determined after freeze-drying at −50 °C for 24 h using a SIM FD5 Series freeze dryer (SIM, Pleasanton, CA, USA).

### 2.4. Determination of Total Lipids and Fatty Acid Profiles

The determination of total lipids and fatty acid profiles was performed as described by Kolouchová et al. [23]. Briefly, lipids were extracted using the Bligh-Dyer method. The extracted lipids were subjected to saponification with 10% (*w*/*v*) potassium hydroxide-methanol solution, followed by methylation with 14% (*w*/*v*) boron trifluoride-methanol solution. Fatty acid methyl esters were analyzed by gas chromatography-mass spectrometry (GC-MS; Thermo Fisher Scientific, Waltham, MA, USA). The GC conditions were as follows: an HP-5 column (60 m × 0.25 mm × 0.25 μm, Agilent Technologies, Santa Clara, CA, USA) was used with a 1 μL injection. Helium was used as the carrier gas at a flow rate of 0.8 mL/min with a split ratio of 1:10. The injector temperature was set at 250 °C, and the detector temperature at 280 °C. The temperature program was as follows: the initial temperature was 140 °C, held for 5 min, then increased by 2 °C/min to 220 °C, where it was held for 20 min. The MS condition included a solvent delay set to 5 min, ionization energy to 70 eV, and a mass range from 35 to 500 amu.

### 2.5. Transcriptomic Analysis

Samples were collected from the negative control (NC) group and the alcohol-treated (ALC; supplementation with 0.5% 1-propanol) group. Total RNA was extracted using the RNA Isolater Total RNA Extraction Kit (Vazyme, Nanjing, China) according to the manufacturer’s instruction. RNA integrity was assessed using the Agilent 2100 Bioanalyzer (Agilent Technologies, Santa Clara, CA, USA). Sample meeting quality criteria (RNA integrity number ≥ 7.0) was used to construct a strand-specific mRNA library with the Hieff NGS^®^ Ultima Dual-mode mRNA Library Prep Kit (Yeasen, Shanghai, China). Finally, the amplified library was sequenced using the Illumina Novaseq 6000 platform (Illumina, San Diego, CA, USA).

The quality-controlled clean reads were mapped to the *Y. lipolytica* CLIB89 reference genome (available at http://ftp.ensemblgenomes.org/pub/fungi/release-62/fasta/fungi_ascomycota3_collection/yarrowia_lipolytica_gca_001761485/, accessed on 20 May 2025) using HISAT2 (version 2.2.1), with gene expression quantified via FPKM. Gene expression analysis was performed using the DESeq2 package (version 3.20) in R (version 3.1), with differentially expressed genes (DEGs) identified based on the threshold of fold change (FC) > 2 and *p* < 0.05. Pearson and Spearman correlation analyses were performed using the Hmisc package (version 4.8-0). The pathway enrichment analyses of DEGs were conducted using the Kyoto Encyclopedia of Genes and Genomes (KEGG; https://www.genome.jp/kegg/, accessed on 25 May 2025) and Gene Ontology (GO; http://geneontology.org/, accessed on 25 May 2025) databases.

### 2.6. Untargeted Metabolomic Analysis

Samples were collected from the NC and ALC groups, followed by untargeted metabolomic analysis using a Vanquish UHPLC system (Thermo Fisher, Waltham, MA, USA) equipped with a Hypersil Gold C18 column (100 mm × 2.1 mm, 1.9 μm; Thermo Fisher, Waltham, MA, USA) coupled to a Q Exactive^TM^ HF-X mass spectrometer (Thermo Fisher, Waltham, MA, USA). The detailed analytical procedures and conditions followed the description by Yuan et al. [24].

Raw data were processed using XCMS (version 3.0) for peak extraction, alignment, and quantification. After normalization, principal component analysis (PCA) and partial least squares discriminant analysis (PLS-DA) were performed using the gmodels (version 2.19.1) and the ropls packages (version 2.18.2), respectively. Based on the PLS-DA results, each metabolite was assigned a variable importance in projection (VIP) score, and metabolites with VIP > 1 and *p* < 0.05 were considered differentially expressed metabolites (DEMs). Pathway analysis of DEMs was conducted using the KEGG database.

### 2.7. Integrated Transcriptomic and Metabolomic Analyses

Based on the expression levels of DEGs and DEMs, 3 models were analyzed, including the KEGG pathway functional model, the two-way orthogonal partial least squares (O2PLS) model, and the Pearson correlation coefficient model.

### 2.8. RT-qPCR

The isolated RNA of *Y. lipolytica* CICC1778 in transcriptome sequencing was reverse transcribed into cDNA. RT-qPCR was conducted as previously described [25,26]. Triosephosphate Isomerase 1 (*TPI1*) was used as a reference gene to normalize cDNA levels in the RT-qPCR assays. Primer sequences were described in Table S1. All primers were synthesized by Tsingke (Beijing, China). Data were analyzed using the relative quantification method (2^−ΔΔCt^).

### 2.9. Statistical Analysis

One-way analysis of variance (ANOVA) was performed using SPSS 20.0 to identify differences. Differences among samples were evaluated using the least significant difference (LSD) test at *p* < 0.05.

## 3. Results

### 3.1. The Characteristics of Growth and Lipid Production of Y. lipolytica CICC1778

**Figure 1:**
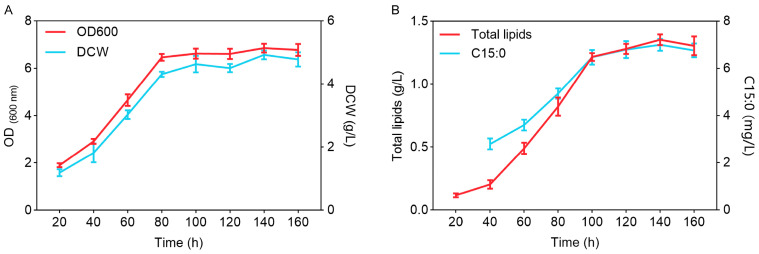
The characteristics of growth and lipid production of *Y. lipolytica* CICC1778. (**A**) The growth curves; (**B**) The production curves of total lipids and C15:0.

In the fermentation medium containing 25 g/L glucose, the growth curves of *Y. lipolytica* CICC1778, as measured by OD_600_ and DCW, exhibited a typical sigmoidal pattern (Figure 1A). It entered the exponential period at 20 h, during which the DCW increased from 1.19 g/L to 4.31 g/L at 80 h, with the OD_600_ reaching 6.47. After 80 h, both OD_600_ and DCW remained essentially constant, indicating that it had entered the stationary phase (Figure 1A). The total lipid content increased rapidly during the exponential phase, from 10% to 19.04%, and subsequently stabilized at above 26% (Figure S1). During the stationary phase, lipid accumulation continued, reaching a maximum of 1.35 g/L at 140 h (Figure 1B). Consistent with this trend, the C15:0 production was low in the early stage of cultivation (2.8 mg/L at 40 h) and then steadily increased, peaking at 7 mg/L at 140 h (Figure 1B).

### 3.2. The Effects of Different Alcohols on the Growth and Lipid Production of Y. lipolytica CICC1778

**Figure 2:**
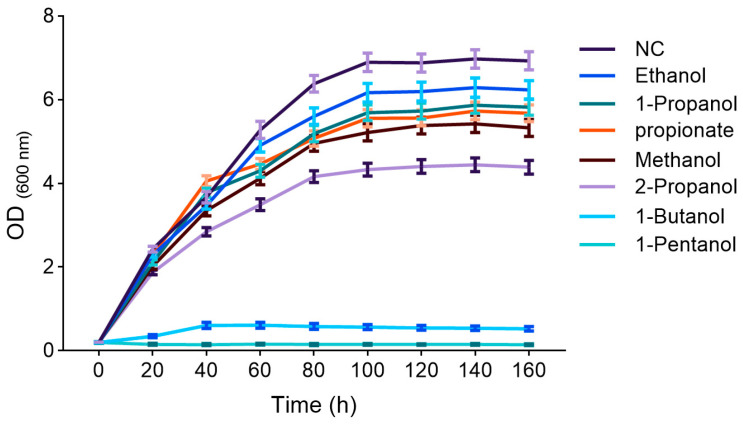
The growth curves of *Y. lipolytica* CICC1778 supplemented with different alcohols. The curves (from top to bottom) correspond to the NC, ethanol, 1-propanol, propionate, methanol, 2-propanol, 1-butanol, and 1-pentanol groups, respectively.

Preliminary experiment revealed that the growth of *Y. lipolytica* CICC1778 was completely inhibited when alcohol was used as the sole carbon source; therefore, the strategy of mixed carbon source was adopted in this study. The effects of different alcohols on the growth of *Y. lipolytica* CICC1778 were shown in Figure 2. The NC group showed the optimal growth kinetics under non-stress conditions, with a final OD_600_ of 6.5–7.2. In contrast, all groups supplemented with alcohols or propionate exhibited different degrees of growth inhibition. Among them, the ethanol group showed the mildest inhibitory effect (stationary phase OD_600_ of 6.17–6.29), whereas 1-propanol and propionate caused similar inhibition, with growth curves consistently below that of the NC group and final OD_600_ stabilizing at 5.56–5.89. The growth curves of the 2-propanol and methanol groups were lower than that of the NC group, while the 1-butanol and 1-pentanol groups exhibited severe growth arrest (OD_600_ < 0.6).

Due to the severe growth inhibition of 1-butanol and 1-pentanol; methanol, ethanol, 1-propanol, 2-propanol, and propionate were selected to investigate their effects on the fatty acid profiles of *Y. lipolytica* CICC1778. As shown in Table 1, unlike the NC group, which exhibited a typical ECFAs-dominated pattern (96.28%), supplementation with 1-propanol and propionate promoted the synthesis of OCFAs (>39%). In the 1-propanol group, the C15:0 content reached 4.89%, which was higher than that in the other groups (*p* < 0.05). Propionate supplementation increased C17:1 content from 0.99% to 42.81% (*p* < 0.05), making it the most abundant fatty acid; however, the C15:0 content in the propionate group was lower than that in the 1-propanol group (*p* < 0.05). Compared with the NC group, supplementation with methanol and ethanol did not alter ECFAs-dominated pattern (>94%, *p* > 0.05).

**Figure 3:**
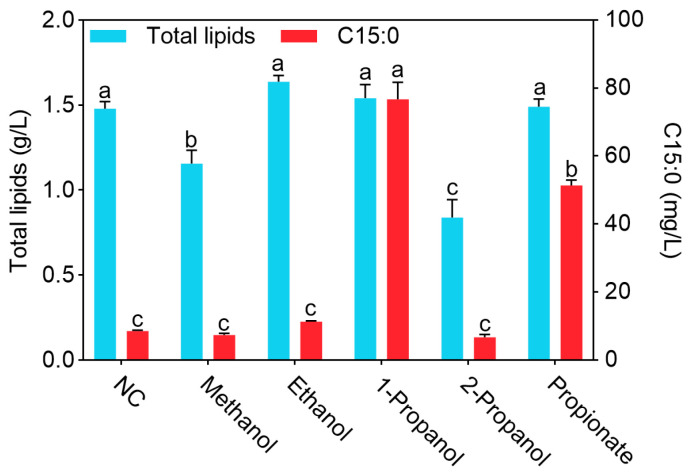
The effects of different alcohols on the lipid production of *Y. lipolytica* CICC1778. Different letters for the same indicator indicated differences (*p* < 0.05). The same below.

Lipid production was shown in Figure 3, the total lipid production in the 1-propanol group reached 1.54 g/L, showing no differences from the NC, ethanol, and propionate groups (*p* > 0.05), but higher than that in the methanol and 2-propanol groups (*p* < 0.05). Methanol and 2-propanol exhibited inhibitory effects on the total lipid production, reducing it to 1.16 g/L and 0.84 g/L, respectively, both lower than that in the 1-propanol group (*p* < 0.05). Regarding C15:0 production, the 1-propanol group achieved the highest level among all groups at 76.68 mg/L (*p* < 0.05), representing a 794.7% increase compared with the NC group. The C15:0 production in the propionate group reached 51.40 mg/L, which was lower than that in the 1-propanol group but higher than that in the other groups (*p* < 0.05). The C15:0 production in the NC, methanol, ethanol, and 2-propanol groups remained at very low levels (<12 mg/L), with no differences among them (*p* > 0.05). Therefore, 1-propanol was used in subsequent studies.

### 3.3. The Effects of Different Concentrations of 1-Propanol on the Growth and Lipid Production of Y. lipolytica CICC1778

**Figure 4:**
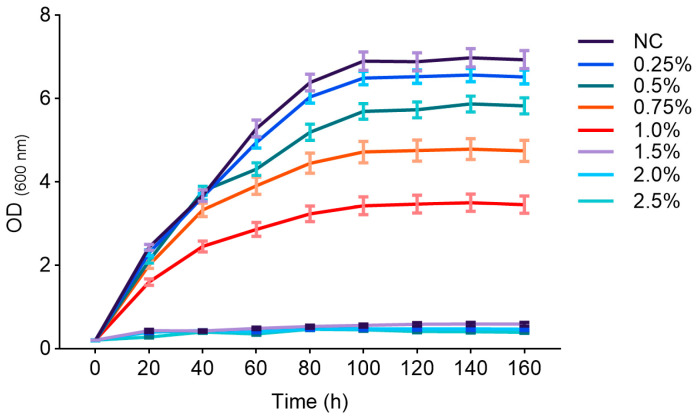
The growth curves of *Y. lipolytica* CICC1778 supplemented with different concentrations of 1-propanol. The curves (from top to bottom) correspond to the NC, 0.25%, 0.5%, 0.75%, 1.0%, 1.5%, 2.0%, and 2.5% groups, respectively.

The effects of different concentrations of 1-propanol on the growth of *Y. lipolytica* CICC1778 were shown in Figure 4. Compared with the NC group (final OD_600_ = 6.89), supplementation with 0.25% and 0.5% 1-propanol reduced the OD_600_ to 6.56 and 5.86, respectively. When the concentration increased to 0.75% and 1.0%, the final OD_600_ decreased to 4.78 and 3.49, respectively. Notably, at concentrations of 1.5% or higher, extreme inhibition was observed, with cell proliferation almost completely arrested and the OD_600_ remaining below 0.6 throughout the entire cultivation period.

Since 1-propanol concentrations of 1.5% or higher caused extreme growth inhibition, the effects of 1% or lower concentrations of 1-propanol on the fatty acid profiles of *Y. lipolytica* CICC1778 were analyzed. As shown in Table 2, compared with the NC group, the C15:0 content increased in all 1-propanol supplemented groups (*p* < 0.05), reaching 5.49% in the 1.0% group. No differences were observed among the groups supplemented with concentrations above 0.25% (*p* > 0.05). The contents of other OCFAs in all 1-propanol supplemented groups were also higher than those in the NC group (*p* < 0.05). In contrast, the ECFAs content decreased progressively with increasing 1-propanol concentration (*p* < 0.05).

**Figure 5:**
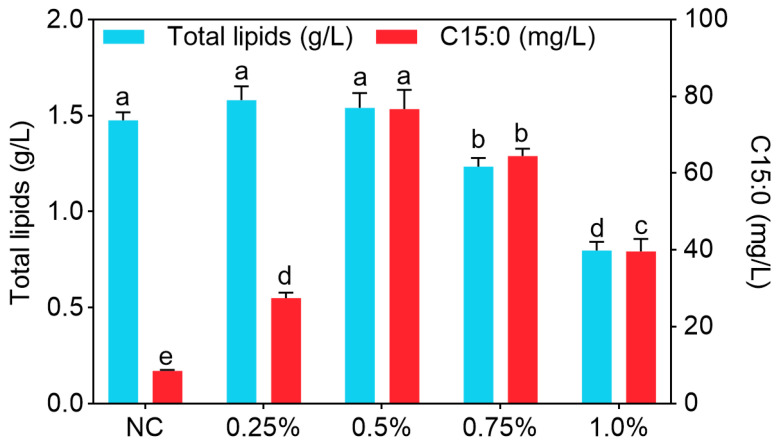
The effects of different concentrations of 1-propanol on the lipid production of *Y. lipolytica* CICC1778. Different letters for the same indicator indicated differences (*p* < 0.05).

As shown in Figure 5, in the groups supplemented with 0.5% or lower concentrations of 1-propanol, lipid production remained above 1.48 g/L and showing no differences among these groups (*p* > 0.05). At concentrations of 0.75% and 1.0%, total lipid production decreased to 1.23 g/L and 0.80 g/L, respectively, both lower than those at concentrations below 0.75% (*p* < 0.05). The C15:0 production in the NC group was at a very low level of 8.58 mg/L. With increasing 1-propanol concentration, the C15:0 production rose sharply, peaking at 76.68 mg/L at 0.5%, which was higher than that at other concentrations (*p* < 0.05). However, when the concentration was further increased to 0.75% and 1.0%, C15:0 production decreased to 64.47 mg/L and 39.63 mg/L, respectively, though both remained higher than that in the NC group (*p* < 0.05).

### 3.4. Transcriptomic Analysis

Transcriptome analysis (Table S2) showed that each sample generated approximately 3.83 × 10^7^ to 4.37 × 10^7^ raw reads. After quality control, the proportion of clean reads exceeded 99.89% for all samples, with Q30 values ranging from 94.08% to 94.81%. Genome alignment analysis indicated that the total mapping rate to the reference genome was 93.43–96.77%, with uniquely mapped reads accounting for the vast majority (93.61%), demonstrating that the sequencing data were of high quality and suitable for subsequent analyses. To evaluate the overall expression profiles among samples, the distributions of FPKM values and pairwise correlations were visualized. As shown in Figure S2, the FPKM values of samples were evenly distributed within each group, with consistent overall trends and no apparent outliers. Spearman correlation coefficients of the samples within the same group were all above 0.95, reflecting highly consistent expression patterns and good reproducibility (Figure S3). 6593 and 6634 genes were identified in the NC and ALC groups, respectively, with 6476 identical genes.

**Figure 6:**
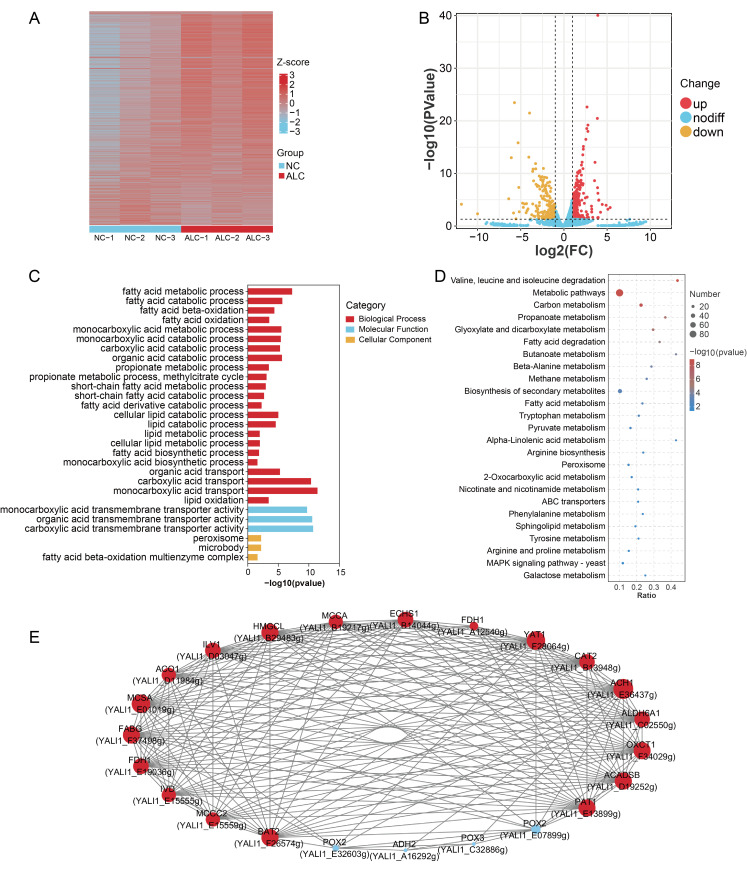
Effects of supplementation with 0.5% 1-propanol on the transcriptome of *Y. lipolytica* CICC1778. (**A**) Heatmap of DEGs between the NC and ALC groups, each with three biological replicates (NC-1, NC-2, NC-3; ALC-1, ALC-2, ALC-3), the color scale represented standardized expression values (Z-scores) across all samples; (**B**) Volcano plot of DEGs between the NC and ALC groups; (**C**) GO enrichment analysis of DEGs; (**D**) KEGG enrichment analysis of DEGs; (**E**) Co-expression network of DEGs related to C15:0 metabolism, the red circles indicated upregulated genes in the ALC group and the blue circles indicated down-regulated genes in the NC group, and the larger size indicated increasing interaction. Gene labels were shown as gene symbols followed by their corresponding locus IDs to distinguish genes with identical symbols. The ALC group was supplemented with 0.5% 1-propanol. The same below.

Using the criteria of *p* < 0.05 and FC > 2, 368 DEGs were identified between the NC and ALC groups, including 221 upregulated and 147 downregulated DEGs (Figure 6A,B, Table S3). GO analysis was performed to functionally classify the DEGs, resulting in 315 enriched GO terms (*p* < 0.05). Among them, 21 GO terms were related to lipid metabolism (Figure 6C). The biological process category included fatty acid metabolic process, fatty acid β-oxidation, fatty acid oxidation, lipid metabolic process, fatty acid biosynthetic process, and propionate metabolic process; the cellular component category included peroxisome and fatty acid β-oxidation multienzyme complex. KEGG pathway enrichment analysis revealed that the DEGs were enriched in 26 pathways (*p* < 0.05) (Figure 6D, Table S4), of which 11 pathways involving 23 DEGs were closely related to C15:0 metabolism (Table 3). Compared with the NC group, the ALC group showed upregulation of Enoyl-CoA hydratase, short chain 1 (*ECHS1*), methylcrotonoyl-CoA carboxylase subunit alpha (*MCCA*), 3-hydroxy-3-methylglutaryl-CoA lyase (*HMGCL*), *ALDH6A1*, acyl-CoA dehydrogenase, short/branched chain (*ACADSB*), putative acyltransferase 1 (*PAT1*), isovaleryl-CoA dehydrogenase (*IVD*), methylcrotonoyl-CoA carboxylase subunit beta (*MCCC2*), branched-chain aminotransferase 2, mitochondrial (*BAT2*), 3-oxoacid CoA transferase 1 (*OXCT1*), *ADH2*, and 3-ketoacyl-(acyl carrier protein) reductase (*FABG*), which were enriched in valine, leucine, and isoleucine degradation, propanoate metabolism, fatty acid metabolism, and fatty acid degradation pathways (*p* < 0.05). In contrast, acyl-CoA oxidase 2 (*POX2*; YALI1_E07899g, YALI1_E32603g) and acyl-CoA oxidase 3 (*POX3*) were downregulated in fatty acid metabolism and fatty acid degradation pathways (*p* < 0.05).

To gain insight into the gene coexpression network associated with C15:0 metabolism, 23 DEGs listed in Table 3 were analyzed. A network constructed from DEG pairs with |r| > 0.80 and *p* < 0.05 revealed 408 interactions among these DEGs (Figure 6E). Acetyl-CoA hydrolase 1 (*ACH1*) exhibited 21 interactions with other genes, while methylcitrate synthase A (*MCSA*) and carnitine O-acetyltransferase *(YAT1)* each showed 21 interactions. In contrast, *POX2* (YALI1_E32603g) displayed 8 interactions, and both *POX3* and *ADH2* exhibited 4 interactions with other DEGs. This map vividly illustrated the relationships among DEGs related to C15:0 metabolism and provided additional information for the study of fatty acid profiles in *Y. lipolytica* CICC1778.

### 3.5. Untargeted Metabolomic Analysis

**Figure 7:**
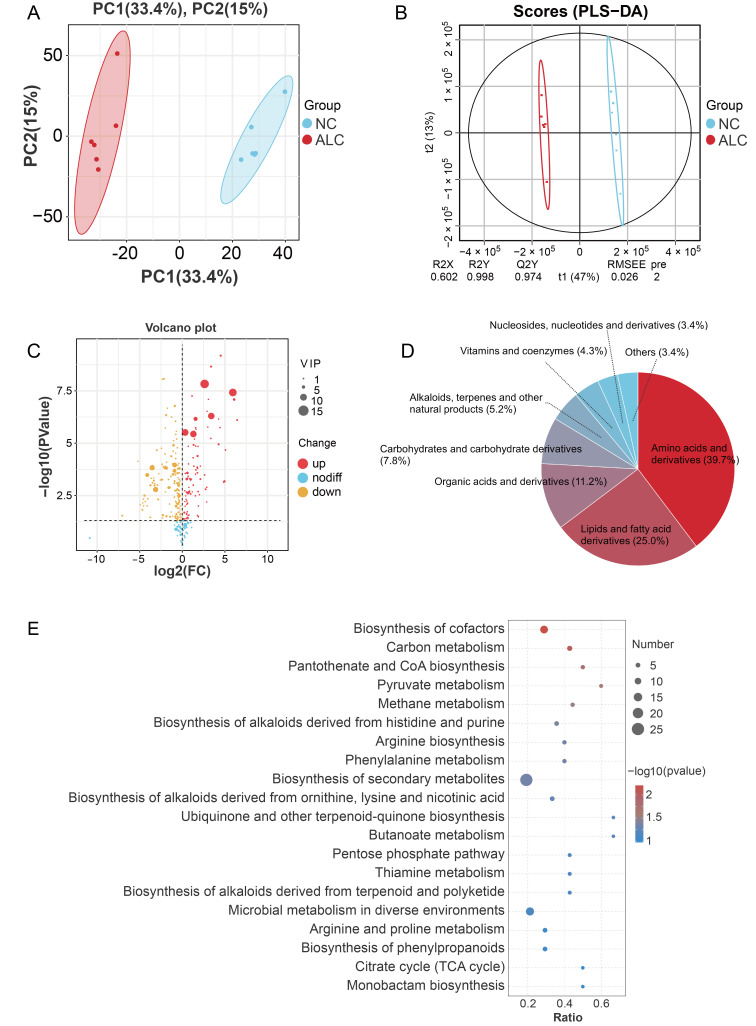
Effects of supplementation with 0.5% 1-propanol on the metabolome of *Y. lipolytica* CICC1778. (**A**) PCA and (**B**) PLS-DA score plot based on metabolites data in the NC and ALC groups; (**C**) Volcano plot of DEMs between the NC and ALC groups; (**D**) DEMs classification; (**E**) KEGG enrichment analysis of DEMs.

Untargeted metabolomics analysis identified 2846 metabolites in the NC and ALC groups, including 1644 metabolites in positive ion mode and 1202 metabolites in negative ion mode. PCA revealed a clear separation between the two groups in principal component space (Figure 7A). The PLS-DA model parameters (R^2^Y = 0.99, Q^2^Y = 0.97) confirmed the high explanatory power and predictive ability of the model (Figure 7B).

Based on statistical analysis and VIP scores obtained from the PLS-DA model, 232 DEMs were identified (*p* < 0.05 and VIP > 1). Compared with the NC group, 89 DEMs were increased and 143 DEMs were decreased in the PLC group (Figure 7C, Table S5). These DEMs were classified into 8 categories: amino acids and derivatives (39.7%), lipids and fatty acid derivatives (25.0%), organic acids and derivatives (11.2%), carbohydrates and carbohydrate derivatives (7.8%), alkaloids, terpenes, and other natural products (5.2%), vitamins and coenzymes (4.3%), nucleosides, nucleotides, and derivatives (3.4%), and others (3.4%) (Figure 7D). KEGG enrichment analysis (Figure 7E) showed that the DEMs were enriched in pantothenate and CoA biosynthesis, butanoate metabolism, pyruvate metabolism, carbon metabolism, and the pentose phosphate pathway (*p* < 0.05).

### 3.6. Integrated Transcriptomic and Metabolomic Analyses

**Figure 8:**
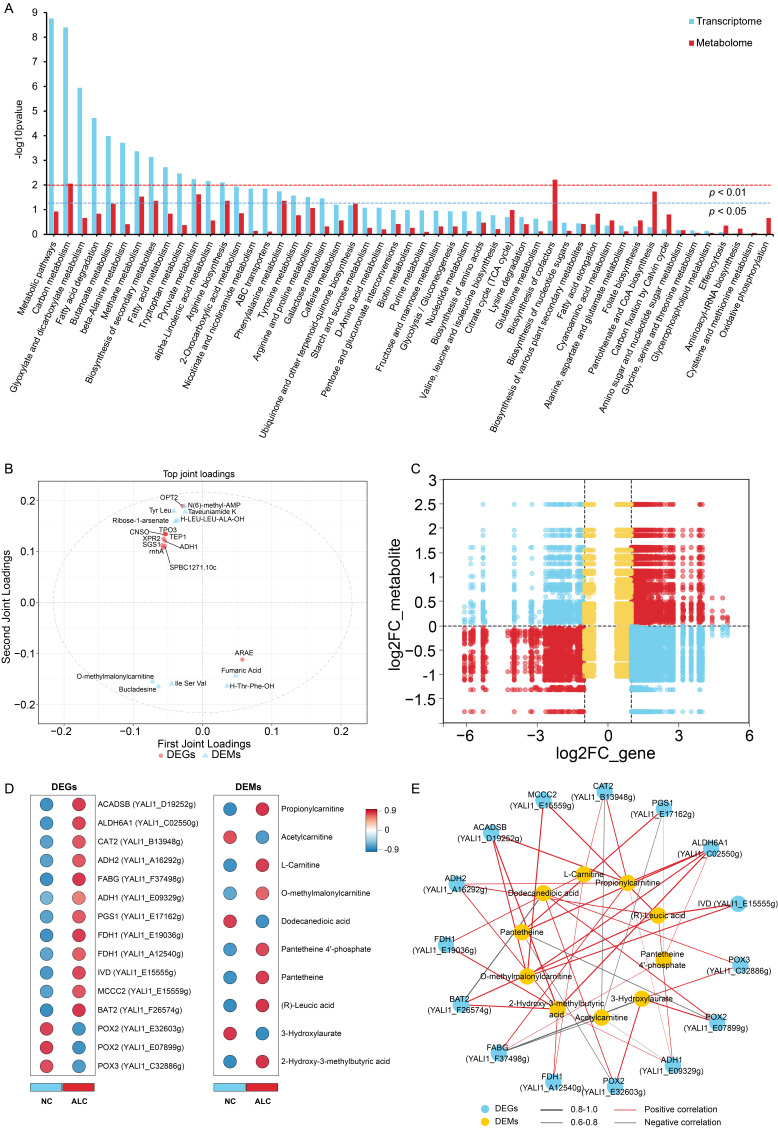
Integrated transcriptomic and metabolomic analyses. (**A**) KEGG pathways co-enriched by DEGs and DEMs; (**B**) Top 10 DEGs and DEMs identified by O2PLS analysis; (**C**) Fold change variations in DEG-DEM pairs with |r| > 0.6, *p* < 0.05; (**D**) Differential analysis of DEGs and DEMs related to C15:0 metabolism; (**E**) Correlation network diagram of DEGs and DEMs related to C15:0 metabolism.

Based on the DEGs and DEMs, a joint KEGG pathway enrichment analysis revealed that 51 pathways were commonly covered, including fatty acid metabolism and degradation, pyruvate metabolism, carbon metabolism, butanoate metabolism, and glyoxylate and dicarboxylate metabolism (*p* < 0.05) (Figure 8A). The O2PLS model explained variance of the correlated part was R^2^Xcorr = 0.93 for the transcriptome and R^2^Ycorr = 0.87 for the metabolome, indicating a pronounced trend of coordinated changes between the two omics layers. Figure 8B showed the top 10 core-associated molecules ranked by loading values, including *ADH1*, O-methylmalonylcarnitine, and isoleucyl-seryl-valine. To comprehensively investigate the mechanism by which 1-propanol regulates the synthesis of C15:0 in *Y. lipolytica* CICC1778, Pearson correlation analysis was performed between the DEGs and DEMs. The results revealed 30,360 DEG-DEM pairs with |r| > 0.6 and *p* < 0.05 (Table S6). Figure 8C showed the fold change variations in these DEG-DEM pairs, with quadrants 3 and 4 representing concordant differential expression patterns, accounting for 32.2%, indicating that the DEGs exerted a positive regulatory effect.

Combined with the differential analysis results (Figure 8D), the Pearson correlation analysis (Figure 8E) showed that propionylcarnitine was positively correlated with *ADH2*, *ADH1*, *FABG*, *ACADSB*, *ALDH6A1*, and carnitine acyltransferase 2 (*CAT2*); O-methylmalonylcarnitine was positively correlated with four key DEGs in branched-chain amino acid catabolism (*IVD*, *MCCC2*, *ACADSB*, and *ALDH6A1*); (R)-leucic acid and 2-hydroxy-3-methylbutyric acid were positively correlated with *IVD*, *BAT2*, *MCCC2* and with *ACADSB*, *ALDH6A1*, *BAT2*, formate dehydrogenase 1 (*FDH1*; YALI1_A12540g, YALI1_E19036g), respectively (|r| > 0.6, *p* < 0.05), indicating an increased carbon flux from branched-chain amino acids and 1-propanol dehydrogenation-oxidation toward propionyl-CoA formation. Regarding the carnitine shuttle, *CAT2* was positively correlated with L-carnitine but negatively correlated with acetylcarnitine, and *FDH1* (YALI1_A12540g, YALI1_E19036g) was positively correlated with L-carnitine and 2-hydroxy-3-methylbutyric acid (|r| > 0.6, *p* < 0.05). In terms of fatty acid oxidation, *POX2* (YALI1_E07899g, YALI1_E32603g) and *POX3* were positively correlated with dodecanedioic acid and 3-hydroxylaurate, whereas *BAT2* was negatively correlated with dodecanedioic acid (|r| > 0.6, *p* < 0.05), suggesting suppression of medium-chain fatty acid (MCFA) oxidation.

### 3.7. RT-qPCR Validation Analysis

**Figure 9:**
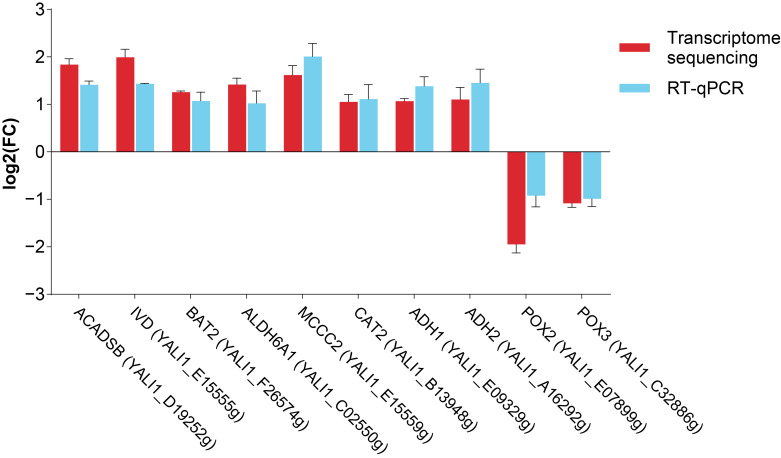
Validation of transcriptome sequencing results by RT-qPCR. Expression levels of 10 key DEGs related to C15:0 metabolism were verified, with the X-axis representing these DEGs. The Y-axis represented the log_2_ fold change in gene expression between the NC and ALC groups.

To validate the reliability of transcriptome sequencing results, 10 DEGs (*ACADSB*, *IVD*, *BAT2*, *ALDH6A1*, *MCCC2*, *CAT2*, *ADH1*, *ADH2*, *POX2* (YALI1_E07899g), *POX3*) were selected for RT-qPCR assay. These DEGs exhibited differential expression between the NC and ALC groups (*p* < 0.05), and the expression trends were consistent with the transcriptome sequencing results (Figure 9). Therefore, the RT-qPCR assay confirmed the reliability of the transcriptome sequencing results.

## 4. Discussion

### 4.1. Effects of Different Alcohols on the Growth and Lipid Production of Y. lipolytica CICC1778

In this study, *Y. lipolytica* CICC1778 exhibited a typical microbial growth and lipid production pattern in glucose fermentation medium. The growth curve followed an S-shaped kinetic profile, and after entering the stationary phase at 80 h, the biomass remained essentially unchanged; however, total lipids and C15:0 continued to be synthesized, reaching their accumulation peaks at 140 h. This trend was consistent with previous observations that oleaginous yeasts, under nutrient-excess but nitrogen-limited conditions, tend to redirect metabolic flux toward lipid synthesis [27,28]. This process reflects metabolic reprogramming of the strain under carbon-nitrogen imbalance, whereby, once cells cease consuming carbon sources for proliferation, more substrates are channeled into fatty acid and triacylglycerol synthesis pathways [29,30]. After entering the stationary phase, the lipid production of *Y. lipolytica* CICC1778 stabilized at over 26%, reflecting a moderate lipid accumulation capacity and providing a suitable baseline for subsequent studies aimed at further increasing C15:0 production through precursor optimization [30].

Previous studies have demonstrated that the synthesis of OCFAs in oleaginous yeasts can be effectively enhanced through regulation by exogenous precursors. Park et al. reported that metabolic engineering of *Y. lipolytica* to strengthen the propionyl-CoA pathway, coupled with propanoic acid supplementation, promoted the accumulation of OCFAs, predominantly C17:1; however, high concentrations of propionate severely inhibited cell growth [7]. Bonzanini et al. compared the fatty acid profiles of different oleaginous yeasts under co-culture conditions with glucose and propionate and found substantial differences in tolerance among these yeasts but a general trend of propionate promoting the accumulation of OCFAs [31]. Multiple reports have indicated that short-chain fatty acids and alcohols exhibited toxicity and growth inhibition in yeasts, whereas one-carbon or two-carbon substrates were better tolerated but can still trigger stress responses and alter lipid unsaturation [32,33,34]. Zhang et al. demonstrated that the addition of 1-propanol to *Rhodococcus opacus* markedly enhanced C15:0 production, revealing the potential of alcohol-based carbon sources as C3 donors [8]. This study showed that both 1-propanol and propionate promoted the synthesis of odd-chain fatty acids, but 1-propanol primarily increased C15:0 production (4.89% and 76.68 mg/L), providing C3 starter units for C15:0 synthesis more effectively. In contrast, propionate redirected the metabolic flux toward C17:1 (42.81%), but the C15:0 production was lower than that in the 1-propanol group, consistent with the findings of Park et al. [7]. Overall, 1-propanol was identified as the optimal exogenous carbon source for efficiently enhancing C15:0 synthesis in *Y. lipolytica* CICC1778.

### 4.2. The Effects of Different Concentrations of 1-Propanol on the Growth and Lipid Production of Y. lipolytica CICC1778

The C15:0 synthesis in *Y. lipolytica* is influenced both by the supply concentration of precursors and by the regulation of the host metabolic network, and high concentrations of C3 precursors are often accompanied by significant growth inhibition. Park et al. evaluated the effects of different carbon sources on OCFAs synthesis in *Y. lipolytica* and found that low concentrations of propionate (2 g/L) could be efficiently utilized, whereas high concentrations (>10 g/L) significantly inhibited growth [7]. Chalabi et al. improved C15:0 production in *Y. lipolytica* by overexpressing Δ9 fatty acid desaturase (*YlOLE1*) and diacylglycerol O-acyltransferase 2 (*YlDGA2*) and by adjusting the ratio of propionate to acetate. Wilbanks and Trinh assessed the toxicity of 32 short-chain organic acids, alcohols, and esters, demonstrating that within the same chemical class, increasing the supplementation level led to a stepwise decrease in growth rate and biomass of *Escherichia coli*, with high concentrations causing complete inactivation [26]. Similarly, Huffer et al. found in comparative studies of yeasts, bacteria, and archaea that alcohol molecules can insert into the lipid bilayer of cell membranes, disrupting membrane fluidity and protein conformation; the higher the concentration, the greater the increase in membrane permeability, which triggered energy metabolism disorders and protein denaturation, ultimately leading to growth arrest [32]. This study used 1-propanol as the C3 precursor source. At low concentrations, 1-propanol caused only mild growth inhibition while promoting the specific accumulation of C15:0, reaching the highest production at 0.5%. When the concentration was increased to 0.75% and 1.0%, total lipids and C15:0 production decreased, suggesting that toxicity effects had begun to impair lipid synthesis; at concentrations ≥1.5%, cells almost completely lost their ability to proliferate.

In this study, the contents of C15:0 and OCFAs in the 1-propanol and propionate groups were consistently higher than those in the other groups. Considering lipid metabolic processes, 1-propanol undergoes sequential oxidation to propionate and is subsequently activated to propionyl-CoA, whereas propionate can be directly converted to propionyl-CoA by propionyl-CoA synthetase [35,36]. The increased availability of propionyl-CoA facilitates the initiation of FAS with a C3 unit instead of acetyl-CoA, thereby driving the synthesis of C15:0 and other OCFAs [9,35]. In contrast, methanol is primarily assimilated through one-carbon metabolism and does not generate propionyl-CoA; ethanol is oxidized to acetaldehyde and acetate, which are further converted to acetyl-CoA; and 2-propanol is mainly oxidized to acetone and subsequently channeled into acetyl-derived pathways [26,32,37,38,39]. These substrates therefore contribute predominantly to acetyl-CoA and ECFAs precursors [9,39]. Therefore, the toxicity pattern of high-concentration 1-propanol toward *Y. lipolytica* and an appropriate concentration of 1-propanol can serve as an efficient precursor source for C15:0 synthesis.

### 4.3. Mechanism of C15:0 Synthetic Regulation by 1-Propanol in Y. lipolytica CICC1778

Transcriptomic and metabolomic analyses revealed a systemic reprogramming of carbon flux distribution and fatty acid synthesis strategies in *Y. lipolytica* CICC1778 under 1-propanol supplementation. In yeast, acyl-CoAs are catalyzed by carnitine acyltransferase (CART) to form the corresponding acyl-carnitine derivatives with L-carnitine. These acyl-carnitines can be efficiently transported among the cytoplasm, mitochondria, and peroxisomes via the carnitine shuttle system, and acyl-CoAs can be regenerated through reverse reactions, thereby buffering transient fluctuations in acyl concentrations and ensuring the supply of substrates required for fatty acid synthesis [40,41,42,43]. Multiple studies have reported that 1-propanol was oxidized to propanal via ADH, subsequently converted to propionate by ALDH, and finally activated to propionyl-CoA by propionyl-CoA synthetase [36,44,45,46]. In this study, propionylcarnitine was positively correlated with *ADH2*, *ADH1*, and *ALDH6A1*, indicating that 1-propanol activates the dehydrogenation-oxidation pathway to generate propionyl-CoA. This pathway complemented the strategy of enhancing propionyl-CoA supply via branched-chain amino acid (BCAA) degradation. Specifically, 1-propanol is dehydrogenated and oxidized by ADH and ALDH to generate propionyl-CoA while reducing NAD^+^ to NADH [47,48,49]. The accumulation of propionyl-CoA disrupts the balance of short-chain acyl-CoAs, and to restore homeostasis, the cell amplifies endogenous pathways capable of supplying propionyl units, among which BCAA degradation is a major source [50,51,52,53]. In addition, propionate derived from the dehydrogenation-oxidation of 1-propanol must be activated to propionyl-CoA by an ATP-dependent acyl-CoA synthetase, thereby increasing the consumption of ATP and free CoA [36,54,55]. The accumulation of NADH together with the altered demand for energy and CoA jointly drives the activation of metabolic pathways that channel electrons into the respiratory chain and generate CoA-linked intermediates [51,56,57]. Within this context, BCAA degradation not only contributes through downstream flavin adenine dinucleotide (FAD)-dependent dehydrogenases such as ACAD and IVD, which transfer electrons via electron-transfer flavoprotein (ETF) to the respiratory chain to oxidize excess reducing equivalents and indirectly regenerate NAD^+^, but also provides CoA-bound carbon skeletons in the form of propionyl units [58,59]. Thus, BCAA flux is enhanced at both the metabolic and regulatory levels, thereby compensating for and restoring carbon-CoA balance as well as redox homeostasis. Crown et al. demonstrated through isotope tracing that the catabolism of valine and isoleucine was an important source of propionyl-CoA in murine adipocytes [53]. Multiple studies have shown that valine, leucine, and isoleucine were transaminated by BAT2 into the corresponding α-keto acids, which were subsequently processed by ACADSB, IVD, MCCC2, and ALDH6A1 to generate propionyl-CoA [53,60,61,62]. In this study, O-methylmalonylcarnitine was positively correlated with *IVD*, *MCCC2*, *ACADSB*, and *ALDH6A1*; likewise, (R)-leucic acid and 2-hydroxy-3-methylbutyric acid were positively correlated with *IVD*, *BAT2*, *MCCC2*, and *FDH1*, indicating that 1-propanol enhanced propionyl-CoA supply by promoting BCAA degradation. Propionyl-CoA then served as the primer in the FAS, condensing with propionyl-ACP to enter the elongation cycle, ultimately synthesizing C15:0 [63].

Messina et al. demonstrated that deletion of the mitochondrial carnitine/acetyl-carnitine carrier 1 (*YlCRC1*) in *Y. lipolytica* altered acyl distribution and increased OCFAs production [41]. For fatty acid oxidation, Mlíčková et al., by knocking out *POXs* in *Y. lipolytica* and performing lipid quantification analysis, demonstrated that *POX* deletion generally increased the storage of neutral lipids in cells [64]. In this study, *CAT2* was positively correlated with L-carnitine and propionylcarnitine, suggesting that 1-propanol may enhance the carnitine shuttle to preferentially transport propionyl-CoA rather than acetyl-CoA, thereby improving substrate supply efficiency for C15:0 synthesis; *POX2* (YALI1_E07899g, YALI1_E32603g) and *POX3* were positively correlated with dodecanedioic acid and 3-hydroxylaurate, indicating that MCFA oxidation was inhibited, which channeled more medium-chain carbon skeletons toward elongation into C15:0.

## 5. Conclusions

Supplementation with 0.5% 1-propanol proved to be an effective strategy for enhancing C15:0 production in *Y. lipolytica* CICC1778. Transcriptomic and metabolomic analyses revealed that 1-propanol underwent dehydrogenation-oxidation and stimulated BCAA catabolism, thereby expanding the propionyl-CoA pool to promote C15:0 synthesis. To the best of our knowledge, this work showed the first report of employing a solvent-engineering approach in *Y. lipolytica* to boost C15:0 production, providing new insights and potential targets for metabolic engineering of C15:0 synthesis.

## Figures and Tables

**Table 1:** The effects of different alcohols on the fatty acid profile of *Y. lipolytica* CICC1778.

Fatty Acids (%)	Groups					
	NC	Methanol	Ethanol	1-Propanol	2-Propanol	Propionate
C15:0	0.58 ± 0.01 ^d^	0.64 ± 0.06 ^d^	0.71 ± 0.03 ^d^	4.89 ± 0.08 ^a^	0.90 ± 0.07 ^c^	3.53 ± 0.05 ^b^
C16:0	15.39 ± 0.12 ^d^	18.50 ± 0.75 ^b^	16.57 ± 0.36 ^c^	16.18 ± 0.11 ^d^	21.85 ± 0.94 ^a^	12.35 ± 0.12 ^e^
C16:1	22.40 ± 0.23 ^a^	19.99 ± 0.61 ^b^	21.68 ± 0.35 ^ab^	7.51 ± 0.08 ^d^	20.43 ± 0.80 ^b^	10.74 ± 0.09 ^c^
C17:0	2.12 ± 0.06 ^d^	2.27 ± 0.11 ^d^	2.31 ± 0.03 ^d^	4.42 ± 0.04 ^a^	2.82 ± 0.15 ^c^	4.00 ± 0.06 ^b^
C17:1	0.99 ± 0.01 ^d^	1.10 ± 0.08 ^d^	1.13 ± 0.03 ^c^	28.00 ± 0.17 ^b^	1.46 ± 0.11 ^c^	42.81 ± 0.16 ^a^
C18:1	29.89 ± 0.34 ^a^	28.32 ± 0.75 ^b^	29.38 ± 0.43 ^ab^	21.68 ± 0.12	28.85 ± 0.65 ^ab^	13.81 ± 0.09 ^c^
C18:2	28.60 ± 0.17 ^a^	29.07 ± 0.56 ^a^	28.77 ± 0.21 ^a^	14.69 ± 0.12 ^c^	23.45 ± 0.98 ^b^	11.54 ± 0.09 ^c^
C19:1	0.03 ± 0.00 ^e^	0.11 ± 0.01 ^d^	0.12 ± 0.01 ^d^	2.64 ± 0.01 ^b^	0.24 ± 0.03 ^c^	1.22 ± 0.01 ^a^
OCFAs	3.72 ± 0.18 ^d^	4.13 ± 0.24 ^d^	4.27 ± 0.08 ^d^	39.95 ± 0.05 ^b^	5.42 ± 0.36 ^c^	51.56 ± 0.07 ^a^
ECFAs	96.28 ± 0.31 ^a^	95.88 ± 0.24 ^a^	95.73 ± 0.08 ^a^	60.06 ± 0.05 ^c^	94.58 ± 0.36 ^b^	48.44 ± 0.07 ^d^

OCFAs, odd-chain fatty acids; ECFAs, even-chain fatty acids; Different letters within the same row indicated differences (*p* < 0.05). The same below.

**Table 2:** The effects of different concentrations of 1-propanol on the fatty acid profiles of *Y. lipolytica* CICC1778.

Fatty Acids (%)	Groups				
	NC	0.25%	0.50%	0.75%	1.00%
C15:0	0.58 ± 0.01 ^d^	2.61 ± 0.06 ^b^	4.89 ± 0.08 ^a^	5.12 ± 0.17 ^a^	5.49 ± 0.43 ^a^
C16:0	15.39 ± 0.12 ^d^	15.74 ± 0.05 ^cd^	16.18 ± 0.11 ^c^	16.93 ± 0.26 ^b^	18.65 ± 0.32 ^a^
C16:1	22.40 ± 0.23 ^a^	14.98 ± 0.07 ^b^	7.51 ± 0.08 ^c^	6.99 ± 0.12 ^d^	6.53 ± 0.23 ^e^
C17:0	2.12 ± 0.06 ^d^	3.25 ± 0.04 d	4.42 ± 0.04 ^c^	4.60 ± 0.07 ^b^	5.21 ± 0.06 ^a^
C17:1	0.99 ± 0.01 ^d^	14.92 ± 0.14 ^c^	28.00 ± 0.17 ^b^	29.82 ± 0.56 ^a^	29.94 ± 0.55 ^a^
C18:1	29.89 ± 0.34 ^a^	25.91 ± 0.11 ^b^	21.68 ± 0.12 ^c^	20.06 ± 0.38 ^d^	19.14 ± 0.69 ^d^
C18:2	28.60 ± 0.17 ^a^	21.16 ± 0.11 ^b^	14.69 ± 0.12 ^c^	13.73 ± 0.39 ^c^	12.29 ± 0.57 ^d^
C19:1	0.03 ± 0.00 ^e^	1.43 ± 0.01 ^b^	2.64 ± 0.01 ^a^	2.82 ± 0.04 ^a^	2.76 ± 0.08 ^a^
OCFAs	3.72 ± 0.18 ^d^	22.21 ± 0.23 ^c^	39.95 ± 0.05 ^b^	42.36 ± 0.83 ^a^	43.39 ± 0.94 ^a^
ECFAs	96.28 ± 0.31 ^a^	77.79 ± 0.23 ^b^	60.03 ± 0.05 ^c^	57.64 ± 0.83 ^d^	56.61 ± 0.94 ^d^

Different letters within the same row indicated differences (*p* < 0.05).

**Table 3:** The pathways related to C15:0 metabolism.

Pathways	Count	*p*-Value	Genes
Valine, leucine and isoleucine degradation	10	<0.01	*ECHS1*, *MCCA*, *HMGCL*, *ALDH6A1*, *ACADSB*, *PAT1*, *IVD*, *MCCC2*, *BAT2*, *OXCT1*
Carbon metabolism	11	<0.01	*FDH1*, *ECHS1*, *ALDH6A1*, *POX3*, *ILV1*, *ACO1*, *MCSA*, *POX2*, *PAT1*
Propanoate metabolism	5	<0.01	*ECHS1*, *ALDH6A1*, *POX3*, *POX2*
Glyoxylate and dicarboxylate metabolism	5	<0.01	*FDH1*, *ACO1*, *MCSA*, *PAT1*
Fatty acid degradation	7	<0.01	*ADH2*, *ECHS1*, *POX3*, *ACADSB*, *POX2*, *PAT1*, *POX2*
Butanoate metabolism	4	<0.01	*ECHS1*, *HMGCL*, *PAT1*, *OXCT1*
Fatty acid metabolism	7	<0.01	*ECHS1*, *POX3*, *ACADSB*, *POX2*, *PAT1*, *FABG*
Pyruvate metabolism	3	0.01	*ADH2*, *PAT1*, *ACH1*
Peroxisome	6	0.01	*CAT2*, *HMGCL*, *POX3*, *POX2*, *YAT1*
2-Oxocarboxylic acid metabolism	3	0.01	*ACO1*, *MCSA*, *BAT2*
Biosynthesis of unsaturated fatty acids	3	0.04	*POX3*, *POX2*

DEGs marked with an underline were upregulated in the ALC group (*p* < 0.05). enoyl-CoA hydratase, short chain 1 (*ECHS1*), methylcrotonoyl-CoA carboxylase subunit alpha (*MCCA*), 3-hydroxy-3-methylglutaryl-CoA lyase (*HMGCL*), acyl-CoA dehydrogenase, short/branched chain (*ACADSB*), putative acyltransferase 1 (*PAT1*), isovaleryl-CoA dehydrogenase (*IVD*), methylcrotonoyl-CoA carboxylase subunit beta (*MCCC2*), branched-chain aminotransferase 2, mitochondrial (*BAT2*), 3-oxoacid CoA-transferase 1 (*OXCT1*), formate dehydrogenase 1 (*FDH1*; YALI1_A12540g, YALI1_E19036g), acyl-CoA oxidase 3 (*POX3*), threonine deaminase/branched-chain amino acid aminotransferase (*ILV1*), aconitase 1, cytoplasmic (*ACO1*), alcohol dehydrogenase 2 (*ADH2*), aldehyde dehydrogenase 6 family member A1 (*ALDH6A1*), methylcitrate synthase A (*MCSA*), acyl-CoA oxidase 2 (*POX2*; YALI1_E07899g, YALI1_E32603g), *ADH2*, 3-ketoacyl-(acyl-carrier-protein) reductase (*FABG*), acetyl-CoA hydrolase 1 (*ACH1*), carnitine acyltransferase 2 (*CAT2*), carnitine O-acetyltransferase (*YAT1*).

## Data Availability

The original contributions presented in this study are included in the article/Supplementary Materials. Further inquiries can be directed to the corresponding author.

## Supplementary Materials

The following supporting information can be downloaded at: https://www.mdpi.com/article/10.3390/biom15111618/s1, Figure S1: The curve of total lipid content of *Y. lipolytica* CICC1778; Figure S2: Violin plot of gene expression distribution in the NC and ALC groups, each with three biological replicates (NC-1, NC-2, NC-3; ALC-1, ALC-2, ALC-3); Figure S3: Spearman correlation analysis of transcriptome data in the NC and ALC groups, each with three biological replicates (NC-1, NC-2, NC-3; ALC-1, ALC-2, ALC-3). The color scale represented Spearman’s correlation coefficients between samples; Table S1: Primer for RT-qPCR; Table S2: Summary of the reads generated from transcriptome sequencing; Table S3: The DEGs Between the NC and ALC groups; Table S4: KEGG enrichment pathways of the DEGs; Table S5: The DEMs between the NC and ALC groups; Table S6: Pearson correlation analysis between DEGs and DEMs.
