# Supplementary material for: Multi-Omics Analysis Reveals 1-Propanol-Induced Pentadecanoic Acid Biosynthesis in Yarrowia lipolytica
Source: Biomolecules. 2025 Nov 18;15(11):1618. doi: 10.3390/biom15111618 (PMC12650647; doi:10.3390/biom15111618)
Supplement: Supplementary file 1 [file biomolecules-15-01618-s001.zip › Figure S2.pdf]

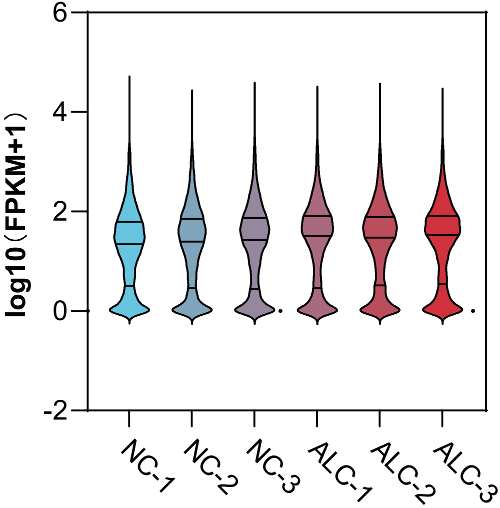

Figure S2. Violin plot of gene expression distribution in the NC and ALC groups, each with three biological replicates (NC-1, NC-2, NC-3; ALC-1, ALC-2, ALC-3).
