# Supplementary material for: Multi-Omics Analysis Reveals 1-Propanol-Induced Pentadecanoic Acid Biosynthesis in Yarrowia lipolytica
Source: Biomolecules. 2025 Nov 18;15(11):1618. doi: 10.3390/biom15111618 (PMC12650647; doi:10.3390/biom15111618)
Supplement: Supplementary file 1 [file biomolecules-15-01618-s001.zip › Figure S3.pdf]

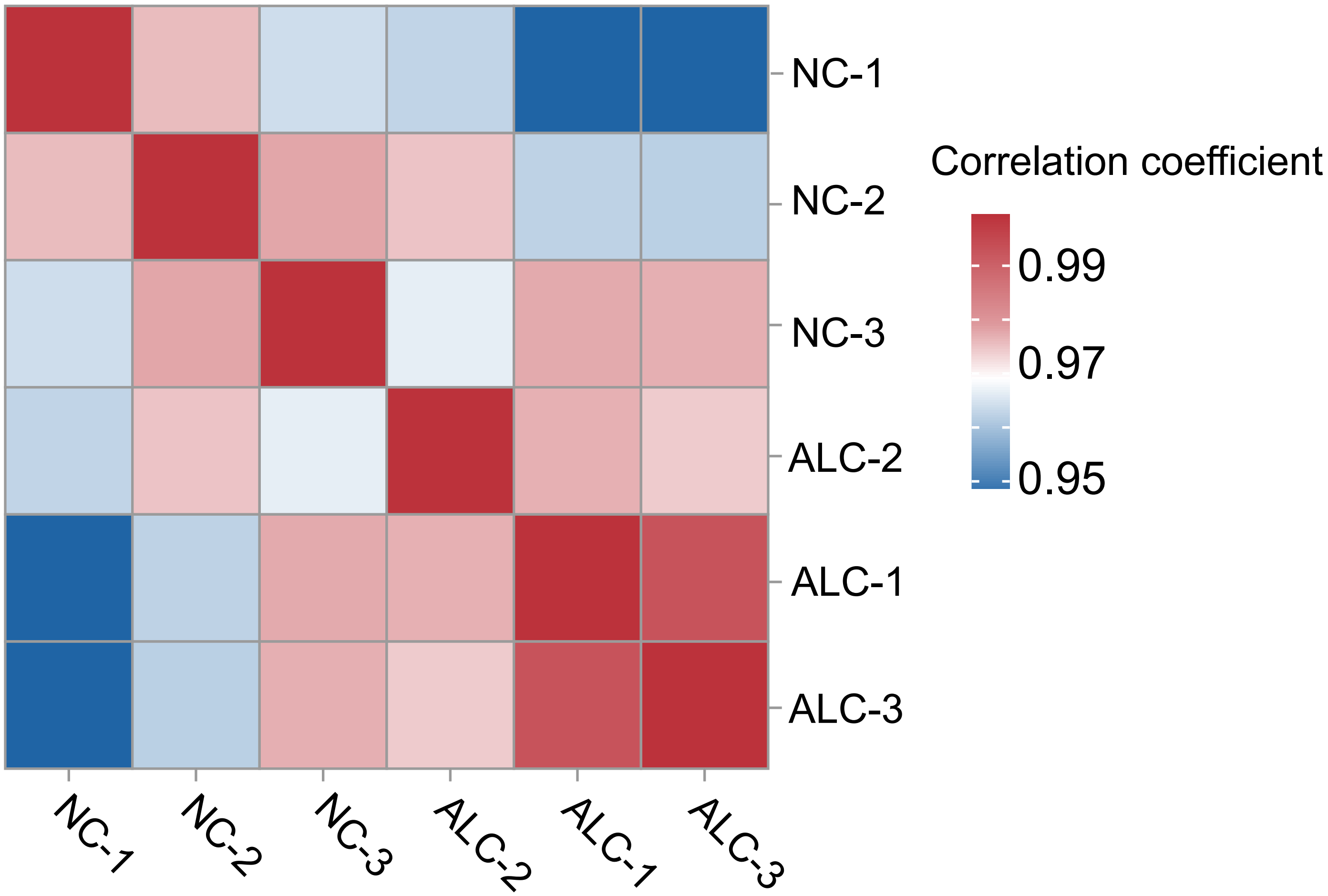

Figure S3. Spearman correlation analysis of transcriptome data in the NC and ALC groups, each with three biological replicates (NC-1, NC-2, NC-3; ALC-1, ALC-2, ALC-3), The color scale represented Spearman's correlation coefficients between samples.
