# Supplementary figures and images for: Multi-Omics Analysis Reveals 1-Propanol-Induced Pentadecanoic Acid Biosynthesis in Yarrowia lipolytica
Source: Biomolecules. 2025 Nov 18;15(11):1618. doi: 10.3390/biom15111618 (PMC12650647; doi:10.3390/biom15111618)

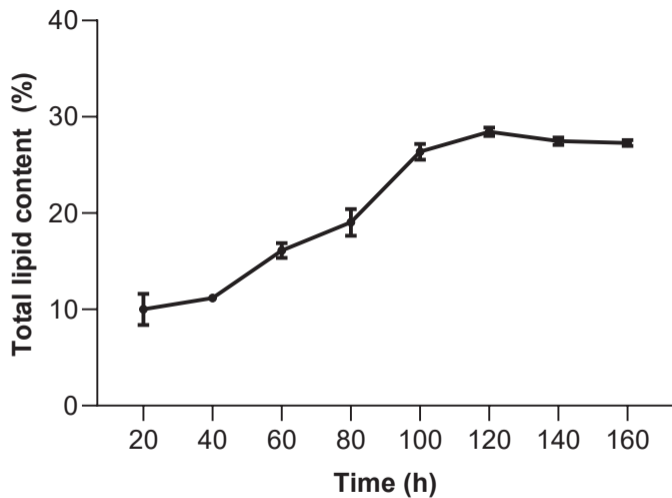

Figure S1. The curve of total lipid content of *Y. lipolytica* CICC1778.

Supplement: Supplementary file 1 [file biomolecules-15-01618-s001.zip › Figure S1.pdf]
